# Prenatal Diagnosis of Sacrococcygeal Teratoma Using Two and Three-Dimensional Ultrasonography

**DOI:** 10.1155/2012/131369

**Published:** 2012-08-09

**Authors:** Livia Teresa Moreira Rios, Edward Araujo Júnior, Luciano Marcondes Machado Nardozza, Antonio Fernandes Moron, Marília da Glória Martins

**Affiliations:** ^1^Mother-Child Unit, University Hospital, Federal University of Maranhão (UFMA), 65085-580 São Luiz, MA, Brazil; ^2^Department of Obstetrics, Federal University of São Paulo (UNIFESP), Rua Carlos Weber, 956 Apartamento 113 Visage, Vila Leopoldina, 05303-000 São Paulo, SP, Brazil

## Abstract

Sacrococcygeal teratoma accounts for half of all fetal tumors, with a prevalence of 1 : 40,000 births. It is believed to originate from pluripotent cells in Hensen's nodule. Although most are benign, they are associated with high morbidity and mortality rates because the fetus develops congestive heart failure and hydrops. Factors leading to poor prognosis include solid components in the mass, and hydrops diagnosed before the 30th week. A case of prenatal sacrococcygeal teratoma diagnosed using B-mode and color Doppler two-dimensional ultrasonography (2DUS) is described, in which three-dimensional ultrasonography (3DUS) enabled characterization of the extent of fetal lesions and allowed the parents to understand the pathological condition better. A 20-year-old primigravida was referred with a solid mass diagnosed in the lumbosacral spine. Examinations performed at our institution revealed pregnancy of 23 weeks and 4 days, with a female fetus presenting a bulky solid mass with cystic components and calcifications, measuring 7.7 × 9.1 × 12.2 cm, starting from the sacral region, with internal flow seen on color Doppler. A new ultrasound confirmed fetal death at 25 weeks and 4 days. Postnatal findings confirmed the diagnosis of sacrococcygeal teratoma. 3DUS can be used in cases of sacrococcygeal teratoma to assess the development of tumor during the prenatal and to allow better understanding of this anomaly by the parents.

## 1. Introduction

Sacrococcygeal teratomas are the commonest retroperitoneal benign tumors. They are believed to originate from pluripotent cells in Hensen's nodule, which is located on the anterior surface of the sacrum or coccyx. Although they constitute more than half of all fetal tumors, their incidence is only 1 : 40,000 live births, with a female-to-male ratio of 4 : 1 [1]. Teratomas are generally limited to the coccyx but may extend into the pelvic and abdominal cavities or develop externally. The classification put forward by Altman et al. [2] describes the surgical anatomy of the tumor, but it does not give any information on the prognosis for the prenatal course. Fetuses with large teratomas present a high perinatal mortality rate, caused by high-output heart failure resulting from arteriovenous communication, with subsequent polyhydramnios and hydrops [3]. 

Advances in ultrasonography have enabled accurate early diagnosis of sacrococcygeal teratomas when they present in the form of cystic, solid, or mixed masses that form starting in the sacral region and protrude toward the perineum or buttocks [4]. However, cystic sacrococcygeal teratomas may be erroneously diagnosed as anterior sacral meningomyelocele, especially when they present as posterior cystic masses [4].

Three-dimensional ultrasonography (3DUS) has been used for diagnosing sacrococcygeal teratomas, and there have been reports in which these tumors have been diagnosed during the first trimester, by means of the surface mode [1, 5, 6], along with reports in which three-dimensional power Doppler has been used to map out the entire vascular architecture of the tumor [4, 6–8].

We present a case of solid-cystic sacrococcygeal teratoma that was diagnosed in the 23rd week of pregnancy by means of two-dimensional ultrasonography (2DUS) in association with color Doppler. We emphasize the main findings from 3DUS in rendering mode.

## 2. Case Presentation

**Figure 1:**
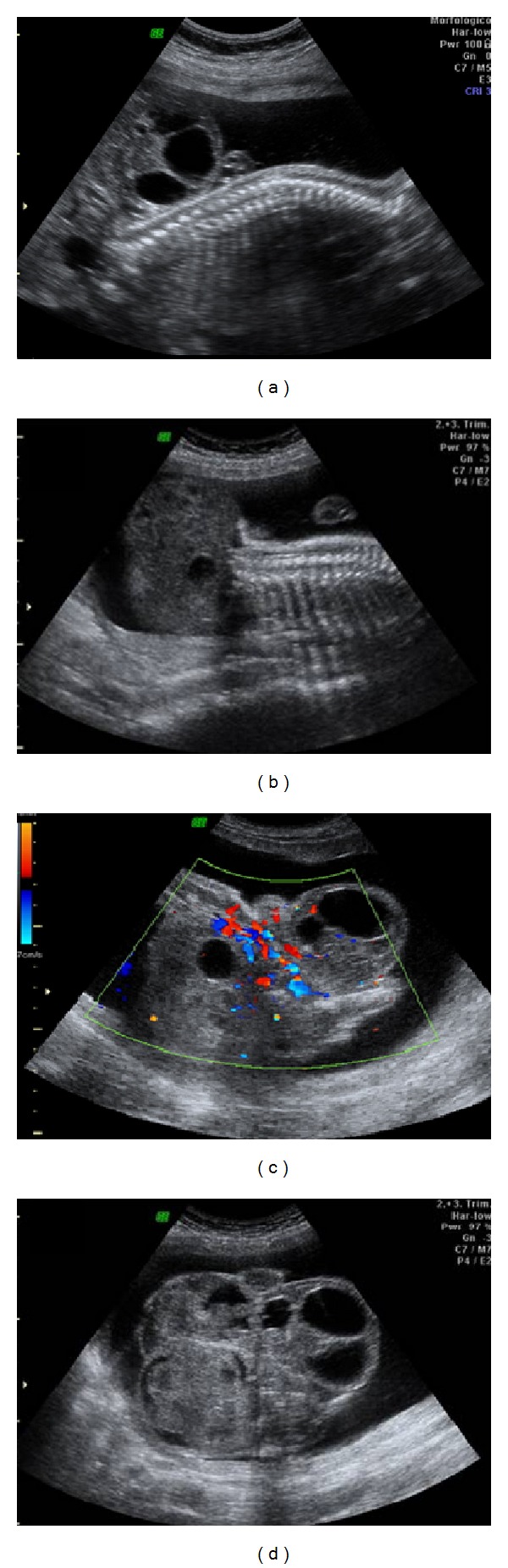
Two-dimensional ultrasound images of a sacrococcygeal teratoma. (a) and (b) Sagittal view of the fetal lumbosacral region, showing a large mass in which solid components predominated, in B mode. (c) Axial view of the fetal pelvis, showing the presence of large-scale flow inside the mass, on color Doppler. (d) Axial view of the fetal pelvis, showing large mass in which solid components predominated, in B mode.

**Figure 2:**
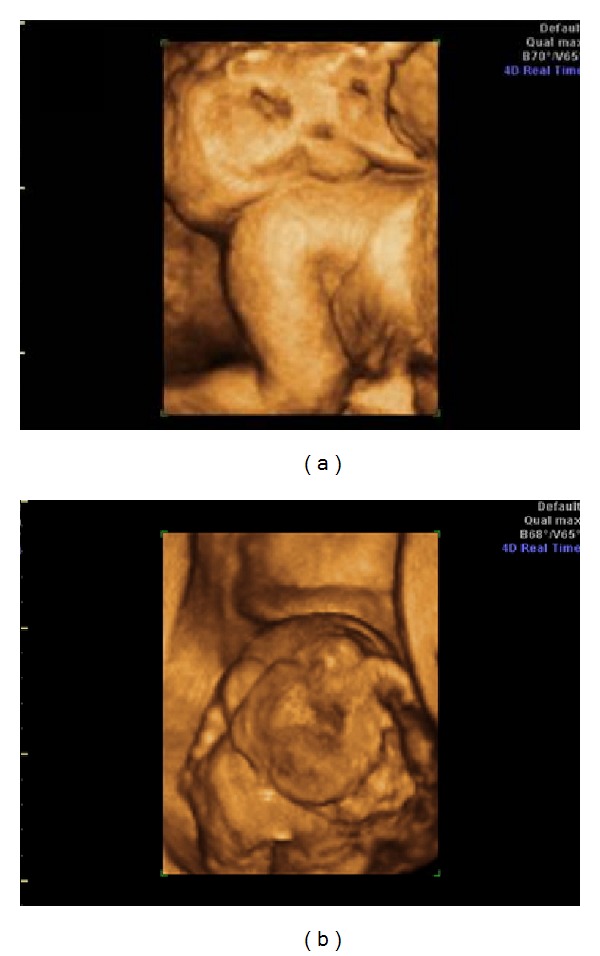
(a) and (b) Three-dimensional ultrasound images of a sacrococcygeal teratoma, in rendering mode, showing the presence of a large mass of lobulated appearance, in which solid components predominated.

**Figure 3:**
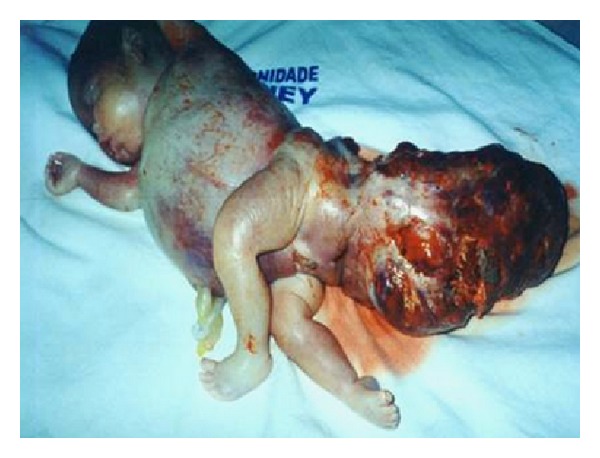
Image of a stillborn fetus showing hydrops and a large sacrococcygeal teratoma in which solid components predominated.

A 20-year-old primigravida was referred to the Gynecology and Obstetrics Service of the University Hospital of the Federal University of Maranhão (UFMA) with a diagnosis of a solid mass in the lumbosacral spine. A 2DUS examination performed in our institution revealed pregnancy of 23 weeks and 4 days, with a female fetus presenting a bulky solid mass with cystic components and calcifications, measuring 7.7 × 9.1 × 12.2 cm, starting from the sacral region, with internal flow seen on color Doppler (Figure 1). 3DUS was performed using the Voluson 730 Pro apparatus (General Electric Medical System, Zipf, Austria), with a volumetric convex transducer (RAB 4–8L) in rendering mode. This clearly showed the spatial relationships between the mass and the fetal pelvis and made it possible for the parents to understand the pathological condition better (Figure 2). A new ultrasound examination confirmed that fetal death occurred at 25 weeks and 4 days of pregnancy, due to hydrops. The postnatal macroscopic findings confirmed the diagnosis of sacrococcygeal teratoma (Figure 3).

## 3. Discussion

Diagnosing sacrococcygeal teratoma prenatally is very important, because large tumors can lead to congestive heart failure, hydrops, and high rates of perinatal mortality. Differentiation between histological types and their potential for malignancy is an important factor in decision making, such as deciding to terminate the pregnancy in countries where this is possible. However, prenatal ultrasonography does not allow such differentiation [9].

In our case, the diagnosis of sacrococcygeal teratoma was made in the 23rd week of pregnancy. With more widespread availability of prenatal screening programs for aneuploidy in the first trimester, it is now possible to make an early diagnosis of sacrococcygeal teratoma [1, 5]. Nonetheless, like in our case, the majority of such cases are referred to specialist services after second-trimester ultrasonography in which a mass in the pelvic region was observed. In our case, the fetus affected by the sacrococcygeal teratoma was female, which is in accordance with the reports in the literature that these tumors occur more frequently in females [1].

In our case, 2DUS showed the sacrococcygeal teratoma as a solid mass with cystic components. Highly vascularized tumors in which solid components predominate are generally associated with immature histology and a higher degree of malignant transformation [9]. These tumors present rapid growth and are associated with high mortality rates due to heart failure and hydrops [6], like in our case, in which the fetus died two weeks after the diagnosis. Hydrops was seen on ultrasound images, probably due to heart failure.

3DUS has been used in a manner that is complementary to 2DUS in diagnosing sacrococcygeal teratoma prenatally [10]. Together with power Doppler, 3DUS makes it possible to map out all of the tumor vascularization, since it has the capacity to pick up signals from small-caliber vessels with low flow velocity, which is very common among neoformed vessels. Furthermore, this method enables identification of communication between these vessels and the fetal circulation [7]. In a case described by Sugitani et al. [4], 3DUS with power Doppler was decisive in differentiating sacrococcygeal teratoma from sacral meningomyelocele sacral, by showing a vessel inside the mass.

In our case, we used 3DUS in rendering mode to evaluate the morphology of the mass and its relationships with the fetal pelvis. 3DUS in rendering mode has already been used in diagnosing two cases of sacrococcygeal teratoma in the first trimester of pregnancy [1, 5]. In the second trimester, there is a single description from Chou et al. [6], of a rapidly progressive immature giant sacrococcygeal teratoma that was diagnosed in the 20th week of pregnancy, in which the newborn died after tumor resection had been performed, which was done after cesarean birth in the 28th week. 3DUS in rendering mode made it possible to view a giant mass of lobulated appearance with a relatively high proportion of solid component, interspersed with cystic areas. The evaluation using 3DUS in that study was comparable with our case, with a high proportion of solid component in relation to cystic component, which is considered to be a poor perinatal prognostic factor.

In summary, we have presented a case of sacrococcygeal teratoma that was diagnosed by means of 2DUS in the 23rd week of pregnancy. 3DUS in rendering mode-enabled assessment of the morphology of the mass and its spatial relationships with the fetal and made it possible for the parents to have a better understanding of the pathological condition. We believe that 3DUS may be an important diagnostic adjuvant to 2DUS in diagnosing sacrococcygeal teratoma prenatally.
